# Microfluidic Generation of Microsprings with Ionic Liquid Encapsulation for Flexible Electronics

**DOI:** 10.34133/2019/6906275

**Published:** 2019-06-19

**Authors:** Yunru Yu, Jiahui Guo, Lingyu Sun, Xiaoxuan Zhang, Yuanjin Zhao

**Affiliations:** State Key Laboratory of Bioelectronics, School of Biological Science and Medical Engineering, Southeast University, Nanjing 210096, China

## Abstract

Inspired by helical or spiral veins, which endow plants with excellent flexibility and elasticity to withstand storms, we present novel hollow microsprings with ionic liquid encapsulation for flexible and stretchable electronics. The microsprings were generated by using a coaxial capillary microfluidic device to consecutively spin poly(vinylidene fluoride) (PVDF) presolution and an ionic liquid, which formed laminar flows in the coaxial injection microfluidic channels. The fast phase inversion of PVDF helps to form the core-shell structure of a microfiber and guarantees the in situ encapsulation of ionic liquid. The hybrid microfiber can then spiral and be further solidified to maintain the helical structure with increasing flow rates of the injection fluids. Because of the feasible and precise control of the injection fluids during the microfluidic spinning, the resultant microsprings have controlled core-shell structures, helical pitches, and corresponding electromechanical properties. By further embedding them into stretchable films, the simple paradigm of a flexible device shows great conductive performance in tensile tests and even motion cycles, which could be explored as a promising candidate in stretchable sensors, flexible electronics, and electronic skins.

## 1. Introduction

Soft and stretchable conductors are essential components in soft robotics as well as wearable, conformable, and deformable electronics [1–5]. Their interconnectors can work similarly to a blood vessel network to transport electrons throughout the whole devices. In comparison with rigid electronic materials, flexible, stretchable, and lightweight conductors are more appealing as they are capable of sensing and conducting under mechanical deformations like tensile strain, bending, and torsion [6–9]. In general, these conductors with different features can be fabricated by integrating conductive agents such as metals, semiconductors, carbonaceous materials, liquid metals, and ionic liquids, into supporting elastic materials, by structural conversion of established brittle and rigid inorganic materials, or directly based on highly stretchable and deformable textile structures [10–14]. Among those conductive agents, ionic liquids have been widely used due to their nonvolatility, low surface tension, relatively low Young's modulus, and good flexibility [15–18]. However, current methods for integrating ionic liquids are based on the sophisticated processes of channel casting, filling, and sealing, which are time-consuming and difficult to realize at small scales. In addition, the resulting ionic liquid products usually have relatively simple structures and are weak at being integrated into flexible systems with much more complex three dimensional structures. Thus, the creation of good stretching and stable conducting materials integrated with ionic liquid is still anticipated.

**Figure 1:**
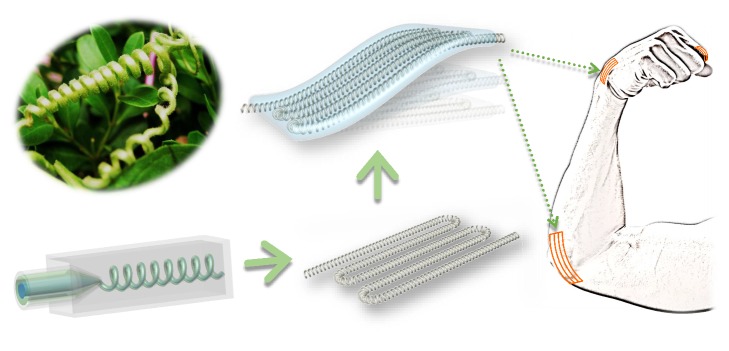
Schematic illustration of bioinspired microspring fabricated by microfluidics for flexible electronics.

In this paper, we present a novel hollow microspring with ionic liquid encapsulation using a microfluidic spinning approach for flexible and stretchable conduction (Figure 1). Helical or spiral veins can endow plants with excellent flexibility and elasticity to withstand storms and these interesting helical structures have stimulated researchers to fabricate materials with such structures. To achieve this goal, many approaches, including chemical assembly, photolithography printing, mechanical twisting, and microfluidics, have been developed to generate the helical structures at nano- or microscales [19–21]. Among these approaches, microfluidic technology has become an outstanding candidate because of its ability to generate materials with various morphologies and functions [22–27]. Feasibility and complexity of channel assembly and construction, precise control on small quantities of fluids in restricted cross-sectional channels, together with various choices of fluids enable the microfluidic spinning technology to be versatile in the creation of microfibers with complex and tunable structures at small scales [28–30]. However, the recent microfluidic spinning approach has not fabricated microsprings with ionic liquid encapsulations, and their potential value in flexible and stretchable conductors remains unexplored.

Here, we employed a coaxial capillary microfluidic device with the function of consecutive generation of hollow poly(vinylidene fluoride) (PVDF) microsprings with ionic liquid encapsulation. Because of the low Reynolds number of the PVDF presolution and ionic liquid, they formed laminar flows in the coaxial injection channels of the microfluidic device. The fast phase inversion of PVDF helps to form the core-shell structure of the microfiber and guarantees the in situ encapsulation of ionic liquid. With increased flow rate of the fluid, the microfiber could then spiral and be further solidified to maintain the helical structure. Because the spinning and spiraling process could be precisely controlled by tuning the phase flow rates and the distance between the outlet tip and the bottom of the collection container, helical microfibers with desired morphologies and microstructures can be continuously generated. As a result, a series of microsprings with different conductivities could be generated and by embedding them into flexible films, they still had great flexibility and electronic performances, showing their practical value for flexible and stretchable conductors. These features proved that the generated microsprings are highly usable in smart flexible electronics.

## 2. Results 

In a typical experiment, a coflow microfluidic system was assembled by coaxially inserting the inner spindle capillary into the outer tapered injection capillary. The generation of core-shell structured microfibers usually depends on the fast and simultaneous gelation along both the inner and outer layers of the precursor flow in microfluidic channels. During a typical spinning process, inner and sheath fluids are introduced into the microfluidic channels to form a coaxially laminar flow due to their small Reynolds numbers. The laminar flow can then be solidified at this stage via phase inversion. The fast reaction can keep the structure of the generated microfibers the same as the laminar flows; thus the microfiber with core-shell structures can be solidified and extruded continuously. Moreover, when there is a flow rate difference between the jetting stream and the surrounding fluid, the stream deforms and begins to spiral in the collection pool. Thanks to the continuous solidification of the extruded microfibers, this helical structure can be fixed and maintained and thus helical microfibers with core-shell structures can be spun continuously.

**Figure 2:**
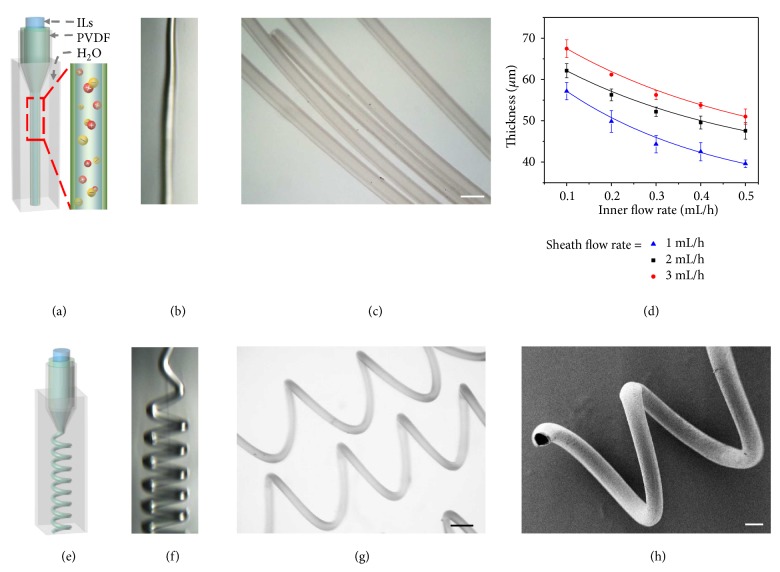
*Microfluidic generation of microfibers*. (a-b) Schematic illustration and real-time microscopy image of microfluidic spinning of straight microfiber with ionic liquid encapsulation, respectively. (c) Optical microscopy image of core-shell structured microfiber. (d) Relationship between thickness of straight microfiber and flow rates. (e-f) Schematic illustration and real-time microscopy image of microfluidic spinning of microspring with ionic liquid encapsulation, respectively. (g) Optical microscopy image of core-shell structured microspring. (h) SEM image of the core-shell structured microspring. Scale bars are 200 *μ*m in (c) and (h), 250 *μ*m in (g), respectively.

In this research, for flexible conduction ability, an ionic liquid (1-ethyl-3-methylimidazolium tetrafluoroborate, [Emim]BF4) was chosen as the core fluid. [Emim]BF4 is a widely used electrolyte and has advantages of nonvolatility, nonflammability. The nonvolatility makes it stable during the microfluidic spinning and spiraling processes together with the following embedding and stretching applications; the nonflammability proves the safe and stable encapsulation amounts of the ionic liquids in microsprings under different weather temperature or animal heats. The electrical conduction of our microfiber depends on the free ions in the encapsulated ionic liquids, which are relevant to their amounts. Thus, the unchangeable amount could stabilize the conductivity of the microsprings. To achieve a uniform shell structure for encapsulation, the sheath phase of PVDF dissolved in N, N-dimethylformamide (DMF) was chosen as the shell material of the microspring because of the rapid phase inversion of DMF induced fast solidification. The inner ionic liquid and sheath PVDF solutions were then simultaneously introduced into the relevant microfluidic channels (Figure 2(a)). The outlet of the microfluidic device was directly immersed into the collection pool containing deionized water. Owing to the hydrodynamic focusing effect and low Reynolds number of the fluids, the immiscible ionic liquid and PVDF fluids hardly mixed with each other. When the three-dimensional coaxial flow was pumping out, the solvent DMF from the PVDF precursor was transferred to the outer deionized water to facilitate the solidification of PVDF; thus the PVDF microfiber can be spun continuously (Figure 2(b)). Because the transfer is very fast, the generated microfibers can replicate the coaxial construction of the microfluidic channels, and the encapsulation of the ionic liquid can take place simultaneously and successfully (Figure 2(c)). As a result, the core-shell structure of the microfibers can be easily tuned by changing the inner and sheath phase flow rates, as shown in Figure 2(d). When the sheath flow rate was fixed, an increase of the inner flow rate brought about a decrease of the shell thickness; conversely, an increased sheath flow rate brought about an increased shell thickness at a fixed inner flow rate. When the increase of the sheath flow continued, the microfiber began deforming and gradually formed a helical structure with a random direction of rotation (Figure 2(e)). The spiraling of the microfiber can be ascribed to the large velocity difference between the microfiber stream and the outer relatively static liquid environment. The outer collection pool also provides enough space for the DMF to transfer to the outer liquid from all directions, which would not interfere with the formation of the helical structure (Figure 2(f)). Finally, the solid microfiber can maintain the helical and core-shell structure owing to the simultaneous process of solidification, spiraling, and encapsulation.

To confirm their structure, the generated helical microsprings were collected for optical observation and dried for scanning electron microscope (SEM) characterization (Figures 2(g) and 2(h)). It was found that they had a free-standing helical geometry and a core-shell structure at the same time. It should be mentioned that the helical microfiber could be uncoiled and recover under strains ([Supplementary-material supplementary-material-1]). Although the dried microfibers under SEM cannot show the inner ionic liquid, it was convincing that the encapsulation was a success from the uniform shell and the continuous spinning and filling process. It could also be proved by the result of infrared spectra ([Supplementary-material supplementary-material-1]). The characteristic peaks of the microfiber (1574 cm^−1^ (framework of aromatic ring), 2984 cm^−1^(C-H), 1185 cm^−1^(C-C)) suggested the successful encapsulation during the spinning process. By tuning the flow rates of the inner and sheath flow rates and the distance between the outlet of the microfluidic device and the bottom of the collection pool, the helical pitches of the solidified microfibers can also be precisely regulated (Figures [Supplementary-material supplementary-material-1] and [Supplementary-material supplementary-material-1]). It can be concluded that the increased phase flow rates and the decreased distance contributed to the decreased helical pitches of the microfibers. Thus, by tuning proper flow rates and the distance between the outlet of the device and the bottom of the collection pool, the core-shell structured helical microfibers with desired morphologies could be continuously spun from this microfluidic method.

**Figure 3:**
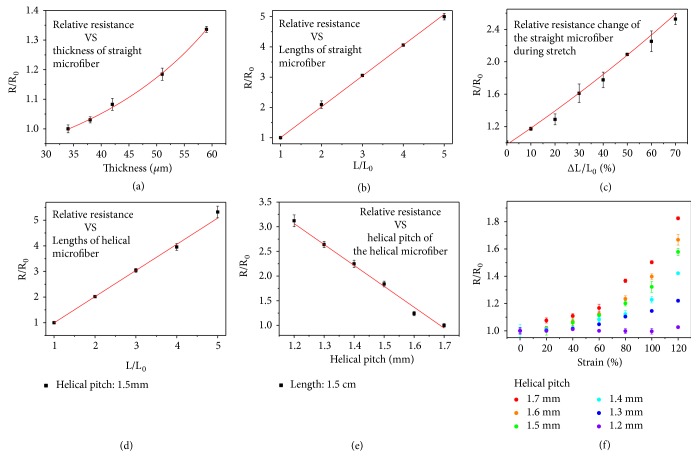
*Conductivity performance of microfibers*. (a) Relationship between relative resistance and the thickness of straight microfiber. (b) Relationship between relative resistance and the length of straight microfiber. (c) Relative resistance change when stretching the straight microfiber. (d) Relationship between relative resistance and the length of microspring. (e) Relationship between relative resistance and the helical pitch of microspring. (f) Relative resistance change when stretching the microspring with different helical pitches.

It should be noted that microfibers with different morphologies can show different conductive performance; thus their relative resistances were firstly measured. The resistance of the straight microfiber conformed to the law of resistance (R = *ρ*_ILs_L/S), where *ρ*_ILs_, L, and S refer to the resistivity of the ionic liquid, length of the microfiber, and the cross-sectional area of the inner core, respectively (Figures 3(a) and 3(b)). The results directly indicated that the longer microfiber showed a larger resistance while a microfiber with a thinner shell showed a smaller resistance. When the shell thickness was fixed, the cross-sectional area was a constant and the increase of the length would bring about a larger resistance; when the length and the diameter of the microfiber remained unchanged, a decrease of the shell thickness resulted in an increased cross-sectional area of the ionic liquid core, which results in a smaller resistance. The smaller resistance corresponded to the better conductivity of the microfiber. This also occurred on helical microfibers with different lengths and helical pitches (Figures 3(d) and 3(e)). For example, the resistance of a microspring with diameter of 100 *μ*m, length of 2 cm, and helical pitch of about 1200 *μ*m is about 1.16MΩ and could be adjusted by further changing its diameter, length, and helical pitch ([Supplementary-material supplementary-material-1]). The resistance of the helical microfiber with a longer length and a smaller helical pitch will be even larger. There are many flexible, bendable, and stretchable textiles that exhibit lower resistance values and they could perform well in sensing applications. However, few of these filaments are generated from such a simple process by introducing conductive agents into microfluidic channels. Thus, the microfluidic generation method will offer inspirations to fabricate conductive microfibers. Also, it is believed that after optimization, the versatile microfluidic technology could generate ionic liquid encapsulated microsprings with much smaller resistance in the near future.

When the fabricated conductive microfibers were stretched, they were also able to respond, as explained by their obvious resistance change as a function of strain. The stiffness of the core-shell structured microfibers was first tested before recording their relative resistance change ([Supplementary-material supplementary-material-1]). It could be easily inferred from the curve that the straight microfiber can only achieve a stretchability of 80%. During the stretching process, the relative resistance change of the straight microfiber (R/R_0_, where R is the resistance in real time and R_0_ is the original resistance) was recorded, as shown in Figure 3(c). The resistance of the 50% stretched microfiber rose to twice as much as that in the original state. The underlying mechanism might lie in the deformation of the core ionic liquid encapsulated channel during the microfiber stretching process. Because the stretching process would cause an elongation of the microfiber and a shrinking of the cross-sectional area due to the Poisson ratio, the resistance underwent an obvious increase during this process. However, the increase of the resistance was not as much in the helical microspring stretching process, especially in those microsprings with smaller helical pitches (Figure 3(f)). This is because the elongation was minimal and negligible at the very beginning of the stretching process; the resistance change mainly depends on the variation of the cross-sectional area of the core ionic liquid encapsulated channel. Until the microspring was stretched to a straight, the increased strain would cause an elongation of the microfiber together with a shrinking of the cross-sectional area, bringing about a remarkable increase in the relative resistance change. Because the microspring with smaller helical pitch had a larger full straight length, the conduction was more reliable during the stretching compared with those microsprings with larger helical pitches. This indicated that the helical microsprings showed enhanced robustness and stretchability in electron conduction under deformation compared with the straight microfibers. The helical structure is the outcome of making full use of the space; thus the small diameter and helical structure guarantee the stable conduction when bended or stretched to a large extent. They will perform more flexibly than conventional bulk materials and are more likely to adapt strict situations like narrow gaps or flexible joints if they were constructed into more complex structures and shapes. They are thus promising candidates for applications in flexible electronics.

**Figure 4:**
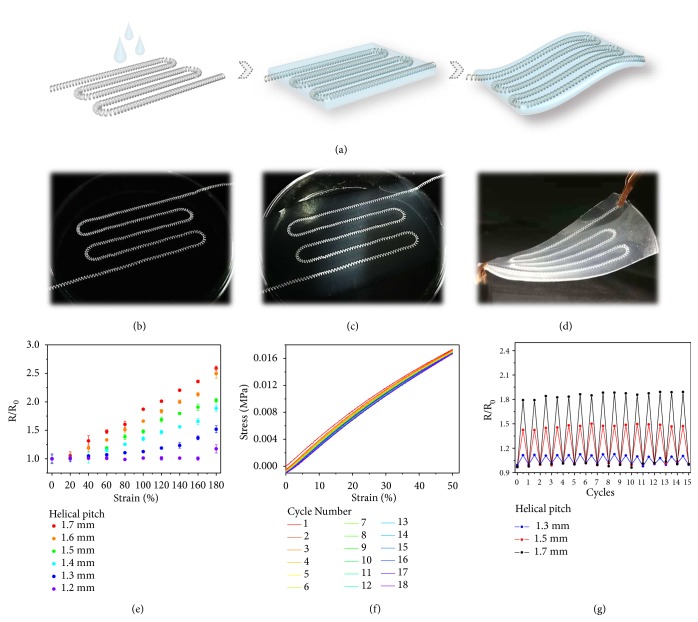
*Generation and characterization of flexible film integrated with microspring*. (a) Schematic illustration of embedding microspring into a flexible polymer film. (b-d) Digital images of the embedding process and the fabricated flexible film. (e) Relative resistance change when stretching the films integrated with microsprings with different helical pitches. (f) Cycled stress-strain test of the composite. (g) Cycled tests of the relative resistance change of the films with microsprings with different helical pitches. The films were all stretched at double length.

To explore the practical value of the generated conductive microsprings, they were then embedded in a flexible polymer film as a paradigm to prototype stretchable electronics (Figure 4(a)). By aligning the helical microfibers as flexible and conductive lines, covering a thin layer of polymer film and curing it, flexible electronics can be achieved (Figures 4(b)-4(d)). The thickness of the film could be adjusted to fit situations requiring thin cross-sections due to the adjustable radius of the microspring by tuning flow rates in microfluidic spinning process. Because the polymer is insulating, the hybrid film could then provide electrical conductivity along the microfiber and insulation along other directions to avoid short-circuiting during further applications. For those embedded microfibers with different helical pitches, their relative resistance change during the stretching process was also studied (Figure 4(e) and [Supplementary-material supplementary-material-1]). The results were nearly consistent with those of a single helical microfiber, which proved that the composition would not interfere with the conductivity of the helical conductors. Thanks to the good stretchability of the film and the helical microfiber itself ([Supplementary-material supplementary-material-1]), plus the low Young's modulus ionic liquid core, the strain-sensing ability functioned steadily even after 18 repeating cycles of stretching and recovering, which can be shown in the cycled stress-strain test (Figure 4(f)). It also suggested that the maximum bending angle of the film was about 130°. Because the helical microfibers were embedded in the film, the elastomeric layer could prevent the embedded microspring from irrecoverable transformations under cycled surpass strains and there was no possibility for the ionic liquid leaking; thus the stability of the helical microwires inside and the potential that the composite could be reused and perform like the original was convinced. This hypothesis can be verified from the cycle test of the relative resistance change of the composite (Figure 4(g)). All of the flexible electronics were treated with repeated strains for 15 times, and the results showed that they can respond reproducibly and reliably to those strains, indicating an outstandingly stable conductivity for practical applications. Additionally, because the ionic liquid is nonvolatile and nonflammable, the complex film could conduct stably at different temperatures ([Supplementary-material supplementary-material-1]).

**Figure 5:**
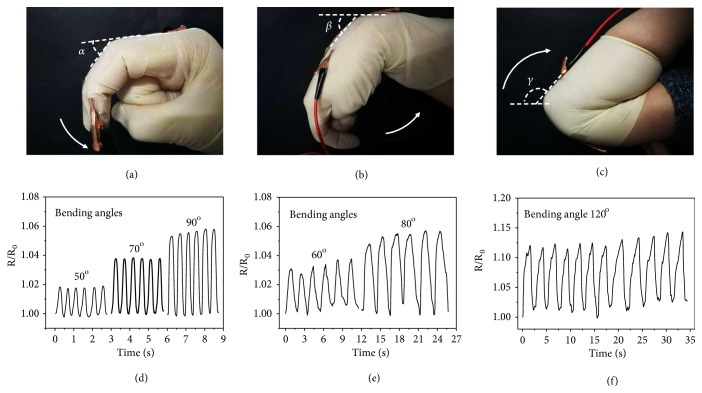
*Conductivity response to various human motions in real time*. (a-c) Digital images of the flexible film responding to bending motions of the (a) finger, (b) wrist, and (c) elbow, respectively. (d-f) Relative resistance change of the flexible film responding to bending motions of the (d) finger, (e) wrist, and (f) elbow at different bending angles, respectively.

Currently, smart flexible devices can display regular motions in real time. An increase in the signal-to-noise ratio is always an object of optimization because of the nonnegligible background noise, which may come from the resistance change of the interconnectors. Thus, the generated ionic liquid encapsulated microsprings showing stable conductivities would be promising candidates in these flexible electronics. To investigate this, the integrated flexible film of the microsprings was employed to detect finger, wrist, and elbow motions, which are common human motions (Figure 5). The film was first stuck on a finger of a glove and worn by a volunteer, and the two terminals containing the integrated microsprings were connected to a digital multimeter, which can record the resistance in real time (Figure 5(a)). When the volunteer bent and straightened his or her finger, the resistances showed corresponding increases and decreases in a very small range and there appeared no changes in a general view (Figure 5(d) and [Supplementary-material supplementary-material-1]). It should also be noted that the resistance changes could manifest the different degree of the finger bending (Figures [Supplementary-material supplementary-material-1]-[Supplementary-material supplementary-material-1]), but there still appeared no changes in the general view. In addition to the fingers, the wrist and elbow are two of the most widely detected motion parts in flexible electronic studies (Figures 5(b), 5(c) and [Supplementary-material supplementary-material-1]). By sticking the integrated film on a wrist or elbow support, the motions can be studied by the corresponding resistance changes (Figures 5(e), 5(f) and [Supplementary-material supplementary-material-1], [Supplementary-material supplementary-material-1]). When showing real-time increases and decreases in a small range, it can still be found that the resistance almost stayed the same in the general view, which indicated that the microsprings can offer relatively reliable conductions in active situations. This was because although bending the finger, wrist, and elbow would strain the film together with the inside integrated microsprings, the deformation at this stage was minimal and would not cause obvious change in resistance of the helical conductors benefiting from their excellent three-dimensional structures. As a result, these microsprings with stable conductivities could then be applied in strain sensors, wearable devices, and flexible electronic fields to decrease the background noise and thus increase the signal-to-noise ratio.

## 3. Discussion 

In summary, we have generated conductive microsprings by using a facile microfluidic spinning approach. The coaxial aligning of the injection capillaries allowed for the formation of the helical microfibers with core-shell structures, which could encapsulate the ionic liquid as conductive agents. Benefiting from the easy manipulation of the phases in microfluidic channels, the shell thickness, helical pitches, and thus the electromechanical properties of the microfibers could be easily adjusted. The microfluidic generation process introduces conductive liquids in microfluidic channels which is seldom involved in previous researches. Also, it is believed that the microfibers with much smaller resistance values could be fabricated after optimization in the near future. The conductive feature of the helical microfibers was then investigated by combining them with stretchable films, and the simple paradigm of stretchable electronics could show great conductive stability and reusability during cycled tensile tests. The accommodation in different motion tests also showed that the conductive helical microfibers are promising candidates in stretchable sensors and flexible electronics. In addition, benefiting from the selectable material compositions, the conductive helical microfibers with ionic liquid encapsulation would be highly usable in different application fields. By constructing them to more complicated structures and shapes, the small diameter microfibers will meet strict requirements for sizes, shape changings, integrating levels, etc., in the future.

## 4. Materials and Methods

### 4.1. Materials

Ionic liquid (1-ethyl-3-methylimidazolium tetrafluoroborate, EMIMBF4) was bought from Aladdin. Polyvinylidene fluoride (PVDF, Mw=534000) was from Sigma-Aldrich. N, N-dimethylformamide (DMF) was purchased from Sinopharm Chemical Reagent Co., Ltd (Shanghai, China). Solutions were all filtered before being pumped into glass microcapillary devices. Ecoflex® 00-30 was purchased from Smooth-On, Inc (Macungie, PA). Water with a resistivity of 18.2 MΩ*∙*cm^−1^ was acquired from a Millipore Milli-Q system. All other chemical reagents were of the best grade available and used as received.

### 4.2. Microfluidics

The capillary microfluidic device was constructed by coaxially aligning one glass capillary with a spindle tip and one with a tapered tip on glass slide. Two capillaries with outer and inner diameters of 1.0 mm and 800 *μ*m (World Precision Instruments) were firstly tapered and sanded to the desired orifice. The orifice of the spindle tip was about 80 *μ*m and that of the tapered tip was about 100 *μ*m. The spindle capillary was then assembled coaxially into the tapered one as the inner flow channel. A transparent epoxy resin was used to seal the capillaries where necessary.

The inner phase was ionic liquid (1-ethyl-3-methylimidazolium tetrafluoroborate, EMIMBF4) and the sheath fluid is PVDF dissolved in DMF at a volume-to-volume concentration of 1:10. A glass vial containing deionized water was put under the outlet of the microfluidic device keeping the liquid covering its tip. The inner flow rates could be changed in 0.1-0.5 mL/h, and the sheath flow rates could be 2-7 mL/h to generated microsprings with different morphologies.

### 4.3. Preparation of Flexible Films Integrated with Conductive Microspring

Firstly the generated microfibers were cut into desired lengths, which were longer than the final tested length to prevent the leaking of the inner ionic liquid. PDMS is also one of the widely used and outstanding elastomeric matrixes, however, in the case of the microfiber with extremely small helical pitch, and could be stretched more than 120%, in which PDMS may not be qualified. As a result, Ecoflex® 00-30 was chosen to produce the elastomeric layer. The two compartments of Ecoflex® 00-30 were mixed in equal volume and homogenized, and the mixture was uniformly coated on the flexibly arranged microfibers. The composites were solidified at room temperature for about 1 hour.

### 4.4. Characterization

The microfluidic spinning process was observed by a fast camera (F032B, Pike, Germany). Bright-field microscopic images were snapped by a stereomicroscope (JSZ6S, Jiangnan novel optics) equipped with a CCD camera (Oplenic digital camera). The cross-sectional microstructure of microspring was characterized by a field emission scanning electron microscope (FESEM, Ultra Plus, Zeiss). The infrared spectra were collected with a Thermo Scientific Nicolet iS50 FTIR spectrometer. The stiffness of the generated microfiber and cycle tests of strain-stress performance of the flexible film integrated with conductive microsprings were characterized by Single Column Table Top Systems (5943, Instron).

The conductivity tests of the generated microfibers were carried out by using a traditional two-probe technique on a semiconductor characterization system (4200-SCS, KEITHLEY). Because the cross-sectional areas of the microfibers were small, the two ends of the microfibers were all firstly coated with the same amount of silver glues to increase the contact area with the probe. A vernier caliper was used to record the strain of the tested microfibers during the stain process. The real-time resistance change was recorded by a digital multimeter (KEITHLEY, USA). Similar to the measurement of microfibers, the two terminal cross-sectional areas of the films were first coated with the same amount of silver glues, covered by conductive tapes, and then connected to the digital multimeter.

## Supplementary Materials

Supplementary Materials Figure S1. The uncoiling and recovery process of microspring. Figure S2. The infrared spectra of ionic liquid, PVDF, and the ionic liquid encapsulated microspring. Figure S3. Relationship between flow rates and helical pitch of microspring. Figure S4. Relationship between height and helical pitch of microspring. Figure S5. The U-I curve of a helical microspring. Figure S6. Stress-strain test of the core-shell structured fiber. Figure S7. Stretching process of the flexible film integrated with microspring. Figure S8. Stress-strain test of the flexible film integrated with microspring. Figure S9. Relationship between the resistance of the flexible film and the temperature. Figure S10. Conductivity response to various human motions in real time.
