# Supplementary material for: Microfluidic Generation of Microsprings with Ionic Liquid Encapsulation for Flexible Electronics
Source: Research (Wash D C). 2019 Jun 19;2019:6906275. doi: 10.34133/2019/6906275 (PMC6750041; doi:10.34133/2019/6906275)
Supplement: Supplementary Materials — Figure S1. The uncoiling and recovery process of microspring. Figure S2. The infrared spectra of ionic liquid, PVDF, and the ionic liquid encapsulated microspring. Figure S3. Relationship between flow rates and helical pitch of microspring. Figure S4. Relationship between height and helical pitch of microspring. Figure S5. The U-I curve of a helical microspring. Figure S6. Stress-strain test of the core-shell structured fiber. Figure S7. Stretching process of the flexible film integrated with microspring. Figure S8. Stress-strain test of the flexible film integrated with microspring. Figure S9. Relationship between the resistance of the flexible film and the temperature. Figure S10. Conductivity response to various human motions in real time. [file 6906275.f1.doc]

Supplementary Materials

**
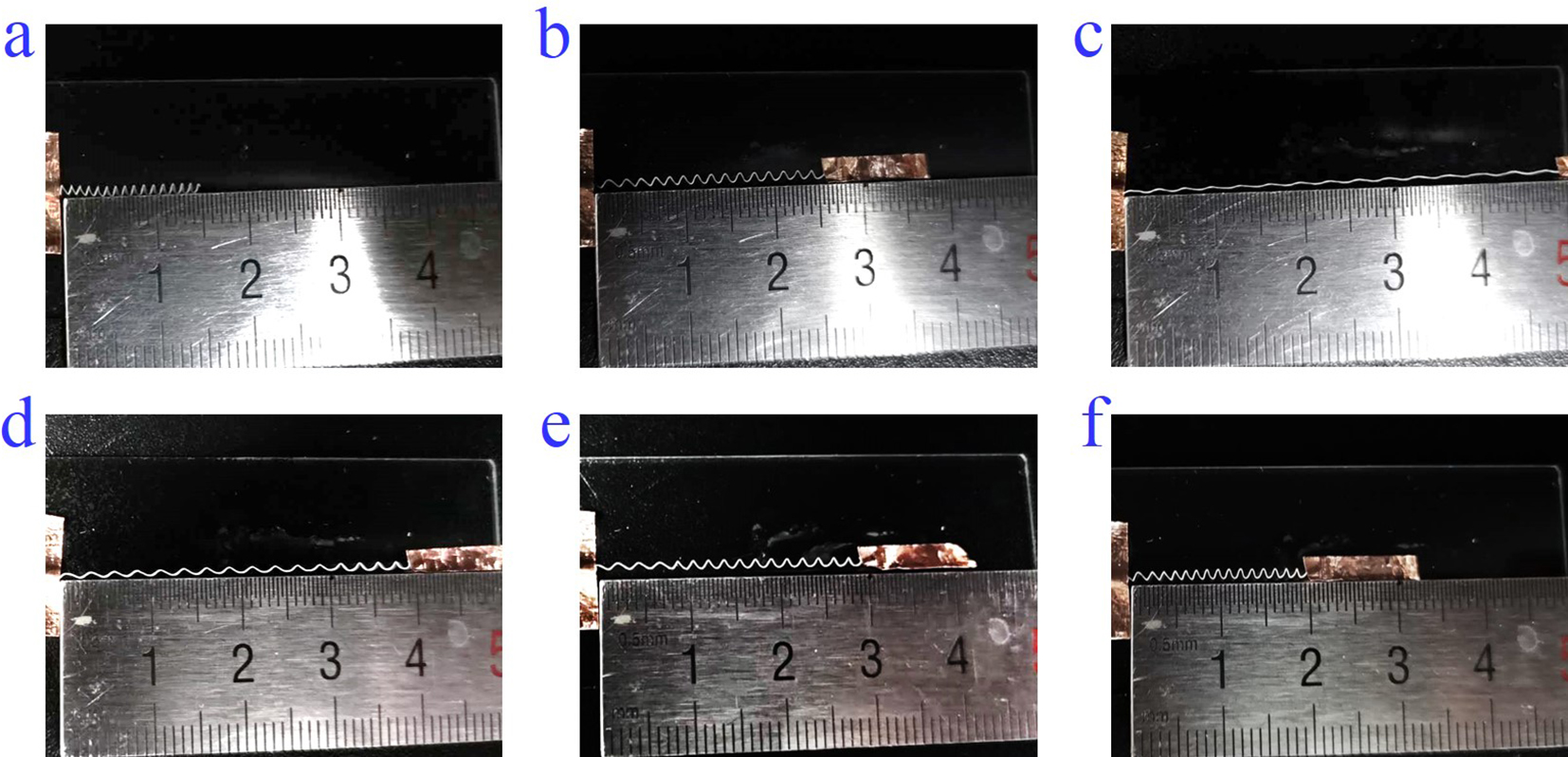
**

**Fig. S1. The uncoiling and recovery process of microspring.** (a-c) The digital images during the uncoiling process of the helical microfiber. (d-f) The digital images during its recovering process.

**
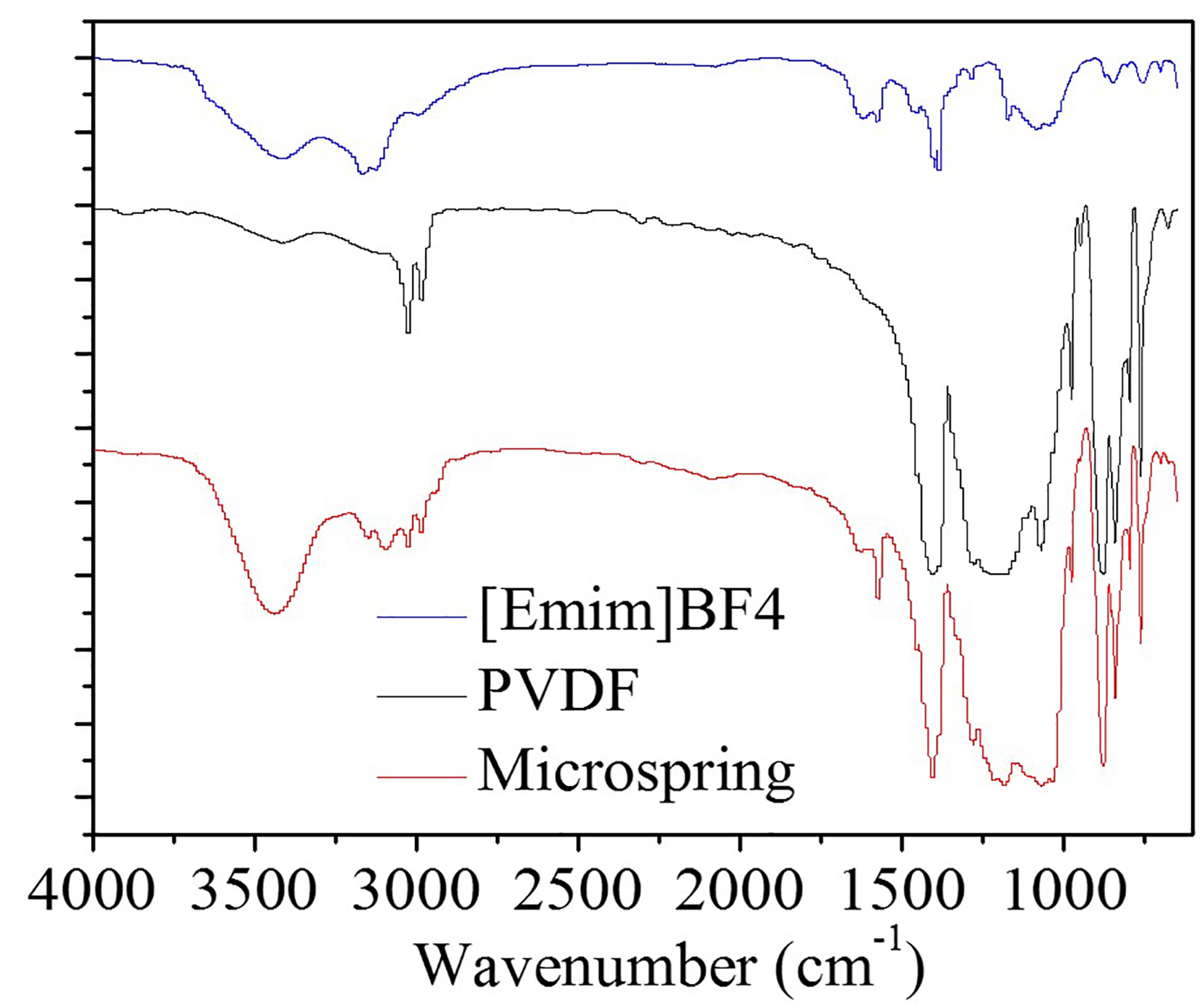
**

**Fig. S2. The infrared spectra of ionic liquid, PVDF and the ionic liquid encapsulated microspring.**


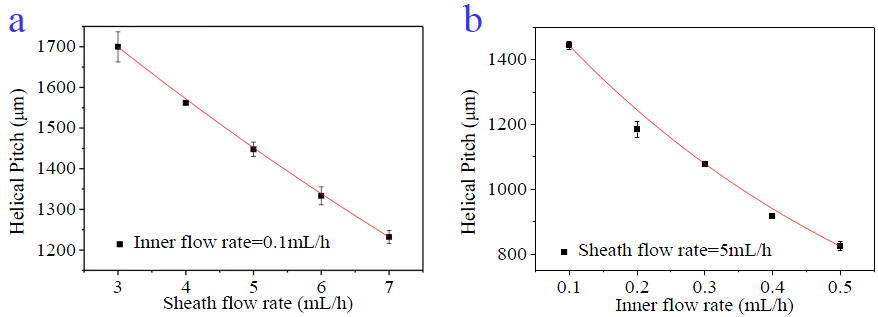


**Fig. S3. Relationship between flow rates and helical pitch of microspring.** (a) Relationship between sheath flow rate and helical pitch. (b) Relationship between inner flow rate and helical pitch.

**
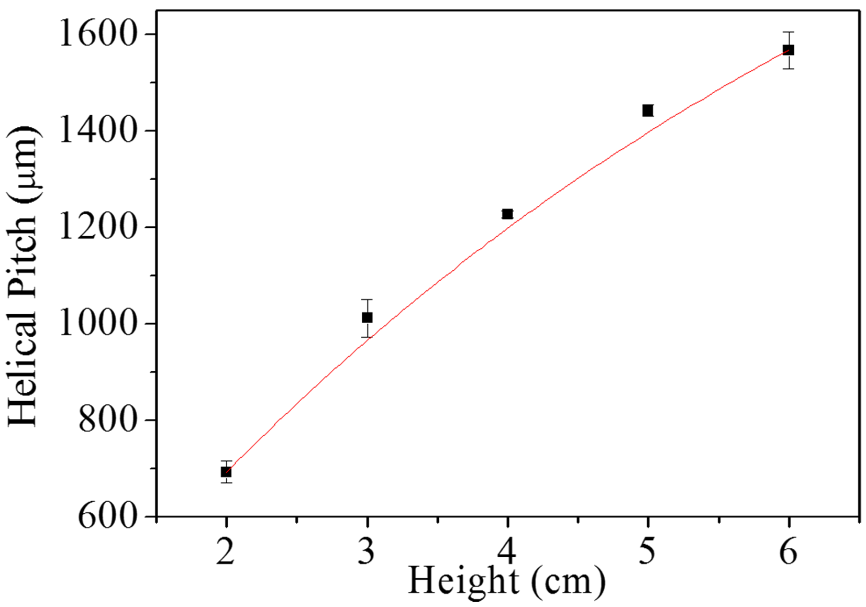
**

**Fig. S4. Relationship between height and helical pitch of microspring.** (The height refers to the distance between the tip of the outlet and the bottom of the collection pool.)


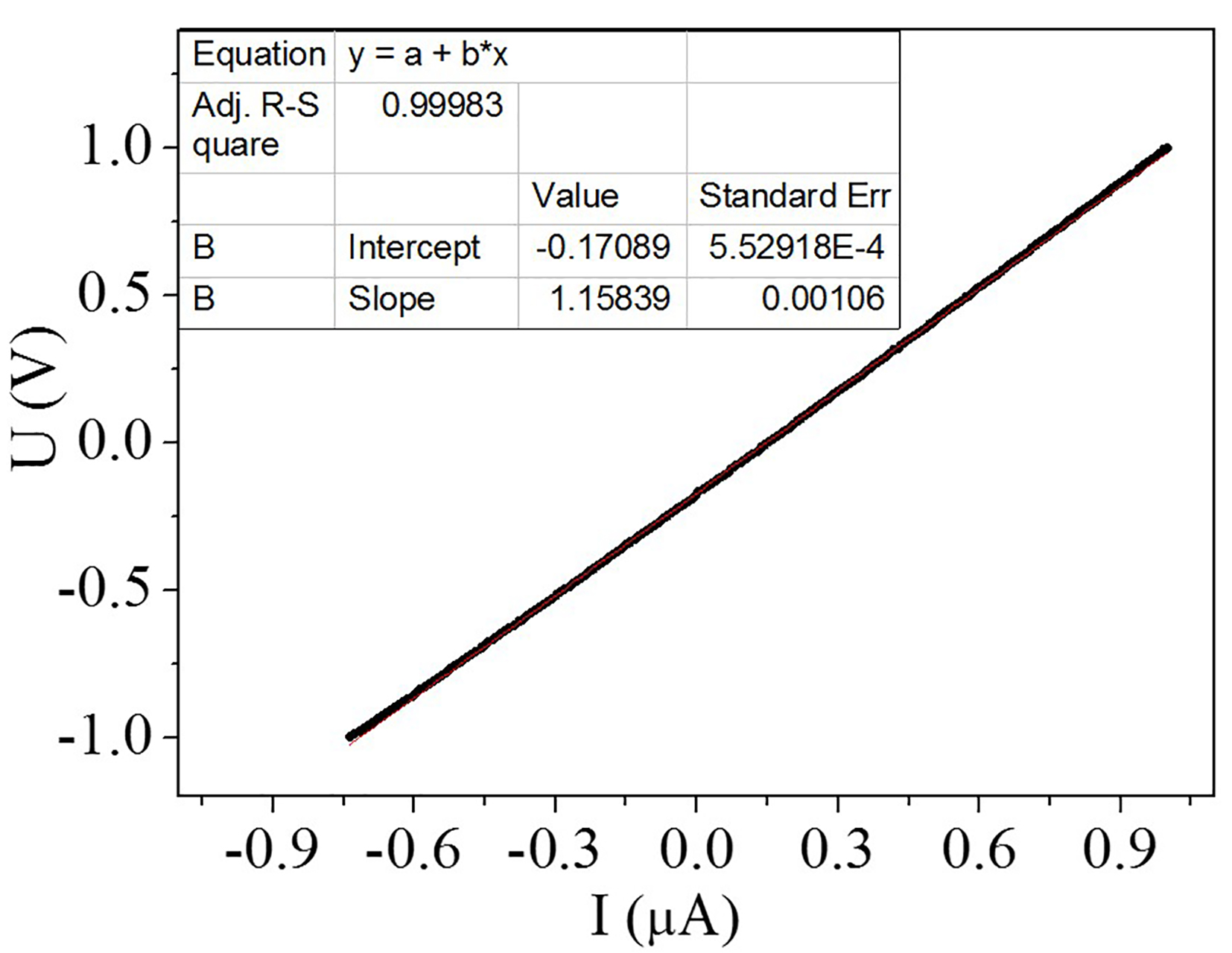


**Fig. S5. The U-I curve of a helical microspring.** (The slope refers to the resistance of the microspring with a diameter of 100μm, length of 2cm and helical pitch of about 1200μm.

**
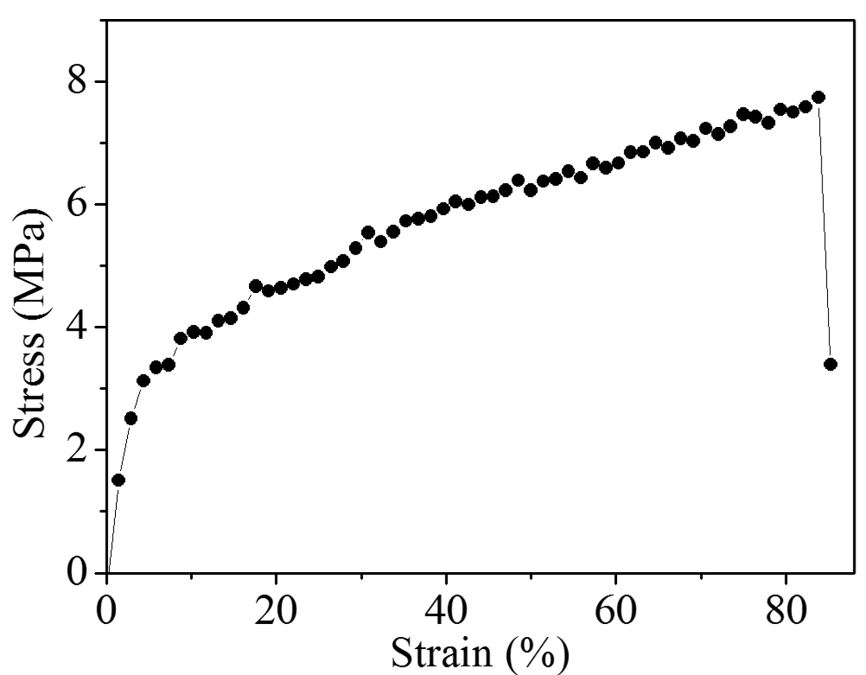
**

**Fig. S6. Stress-strain test of the core-shell structured fiber.**

**
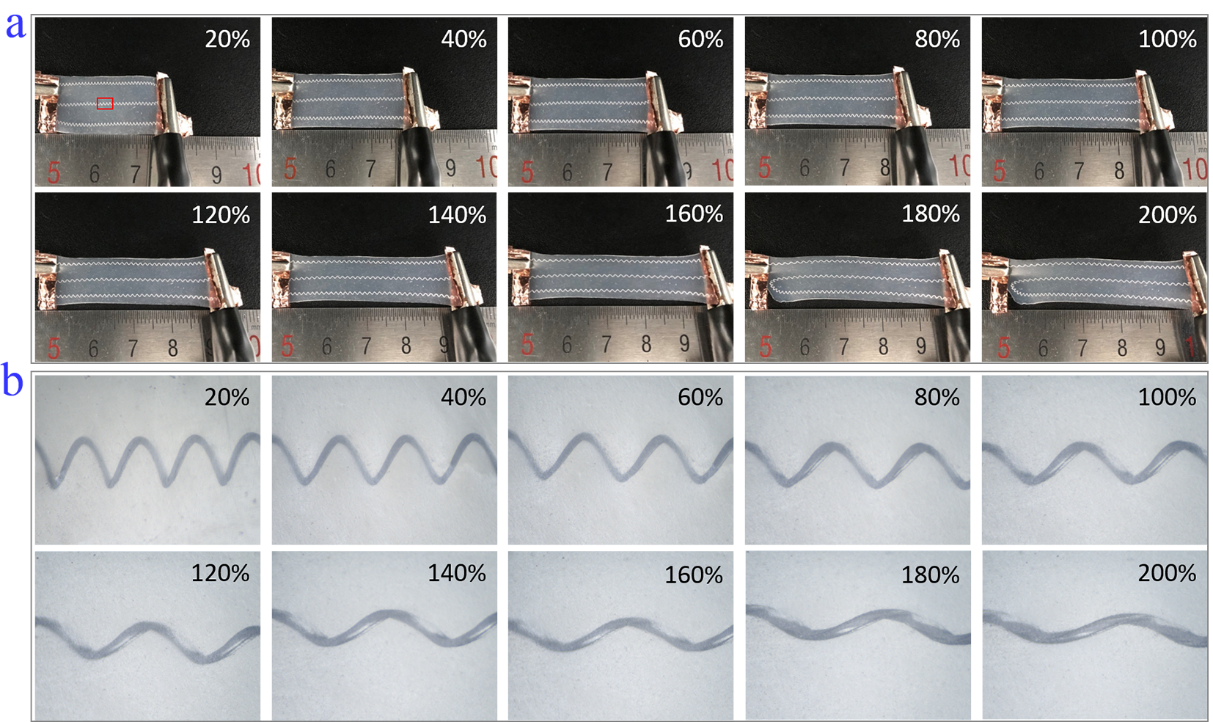
**

**Fig. S7. Stretching process of the flexible film integrated with microspring.** (a) Digital images showing flexible film at different strain state. (b) Optical microscopy images of the shape change of the embedded microspring at different strain state.

**
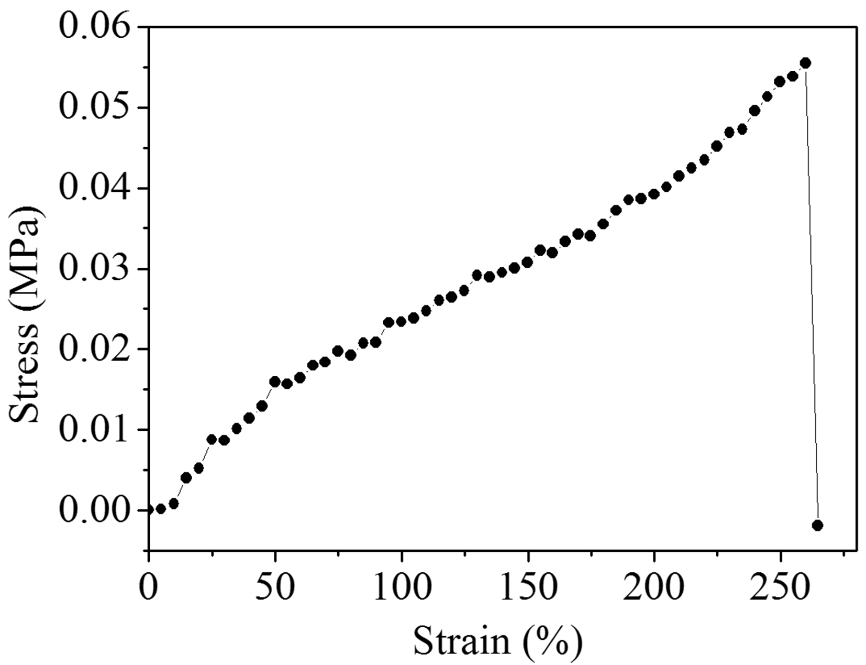
**

**Fig. S8. Stress-strain test of the flexible film integrated with microspring.**

**
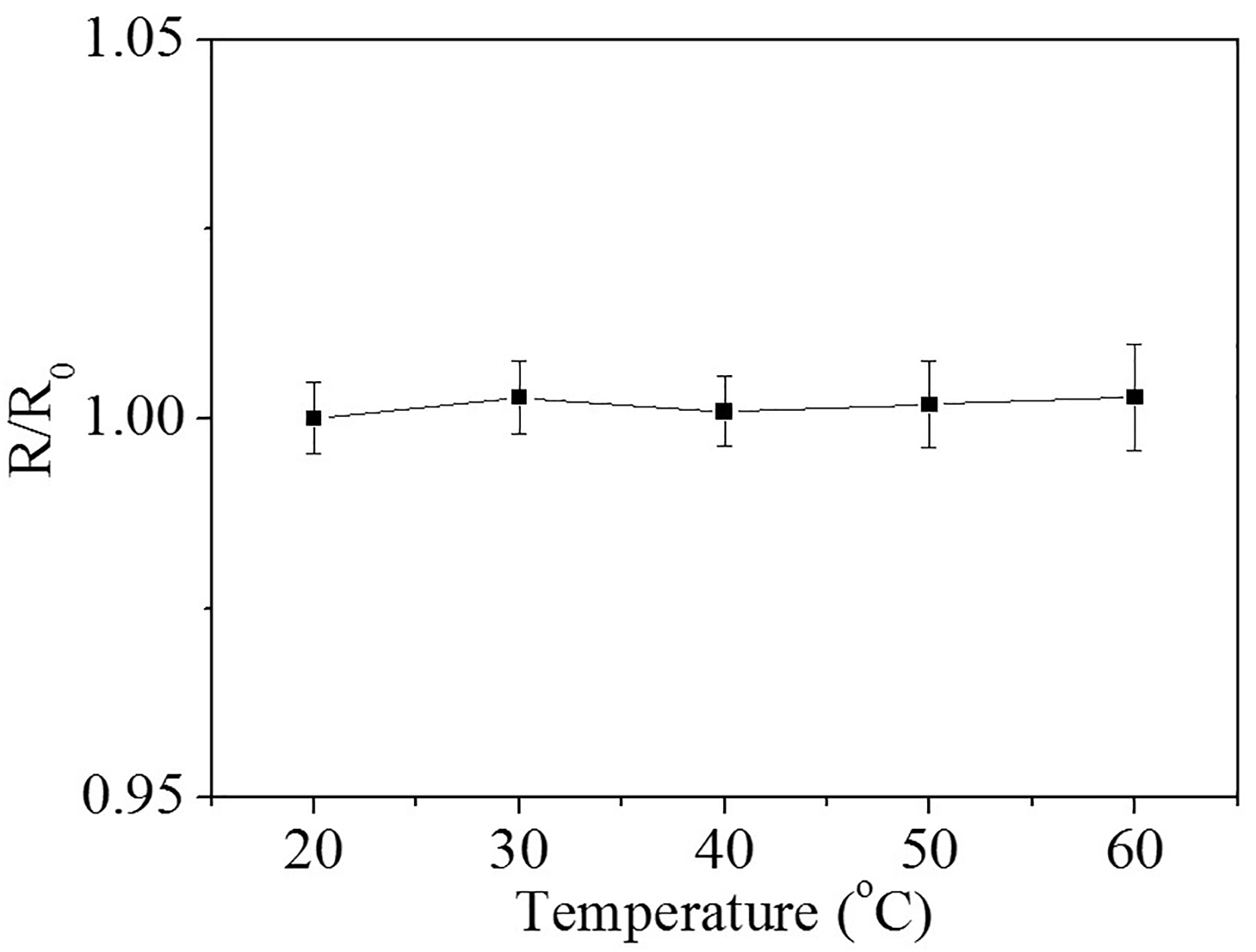
**

**Fig. S9.** Relationship between the resistance of the flexible film and the temperature.


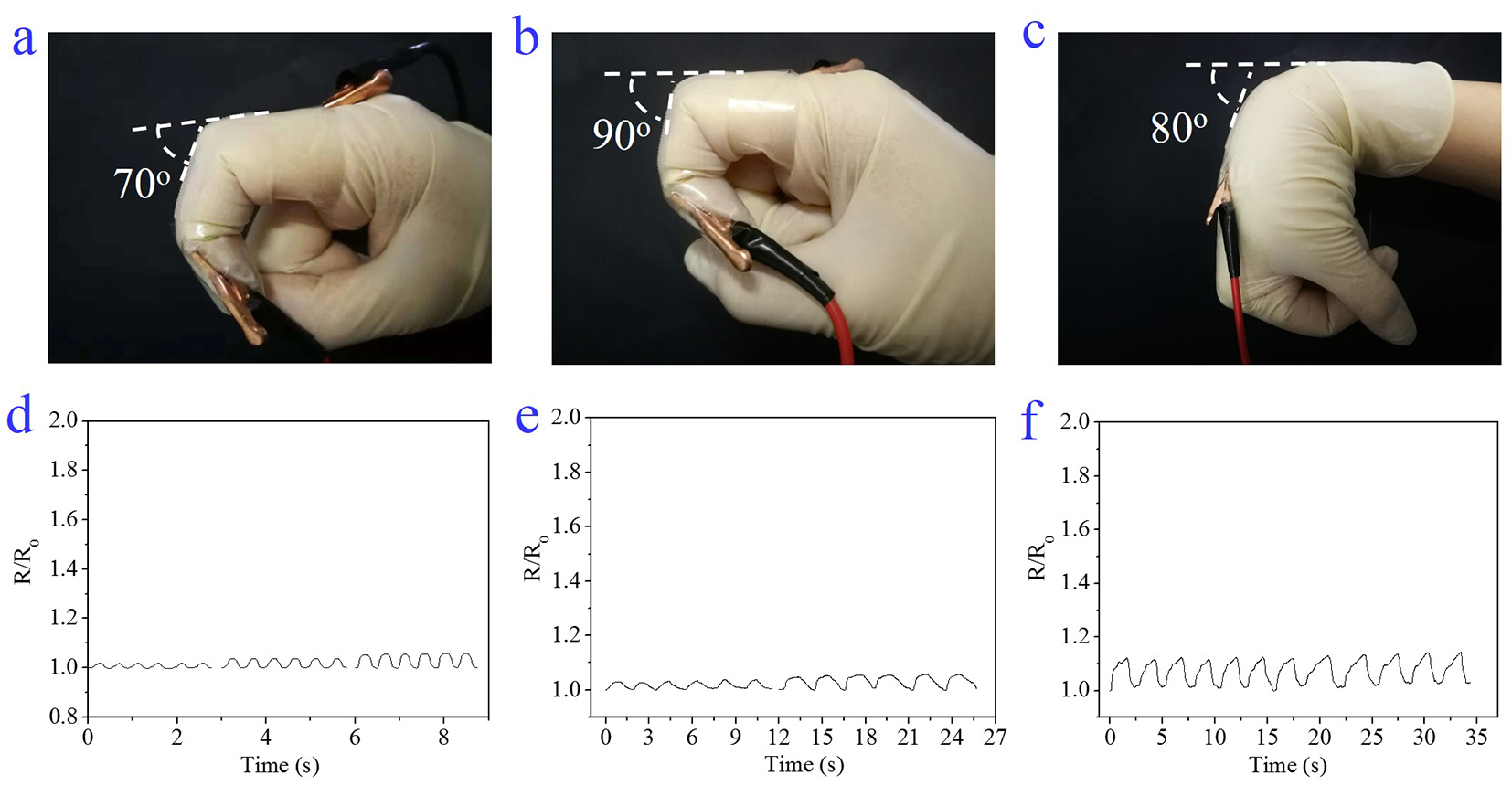


**Fig. S10. Conductivity response to various human motions in real time.** (a-c) Digital images of the flexible film responding to bending motions of the (a-b) finger, and (c) wrist at bending angle of 70^o^, 90^o^, 80^o^, respectively. (d-f) General view of relative resistance changes of the flexible film responding to bending motions of the (d) finger, (e) wrist, and (f) elbow at different bending angles, respectively.
